# Ameliorative Effects of Vitamin C on Heat Stress-Induced Testicular Dysfunction in Wistar Rats

**DOI:** 10.5935/1518-0557.20260015

**Published:** 2026

**Authors:** Damilare Rotimi, Adebanke Olaide Adesupo, Marvis Ayoola, Tomilola Debby Olaolu, Oluyomi Stephen Adeyemi

**Affiliations:** 1 Department of Biochemistry, Landmark University, Omu-Aran 251101, Nigeria; 2 Department of Pediatrics, University of Arizona College of Medicine, Phoenix, AZ, United States; 3 Department of Physical Medicine and Rehabilitation, University of Missouri, Columbia, MO, United States; 4 Tulane University, School of Medicine, Department of Pathology and Laboratory Medicine, New Orleans, Louisiana, 70112; 5 Department of Biochemistry, Medicinal Biochemistry & Toxicology Laboratory, Bowen University, Iwo 232101, Nigeria

**Keywords:** Ascorbic acid, climate change, fertility, oxidative stress, thermal stress

## Abstract

**Objective:**

To evaluate the effects of repeated heat exposure on testicular function and sperm quality in Wistar rats and to determine whether vitamin C mitigates heat stress-induced reproductive damage.

**Methods:**

Twenty-four Wistar rats were allocated to four groups and treated for 28 days: (i) control, (ii) heat exposure (40°C for 4 hours/day), (iii) vitamin C (50 mg/kg), and (iv) vitamin C plus heat exposure. Antioxidant status (superoxide dismutase, catalase, reduced glutathione), oxidative/nitrosative stress markers (malondialdehyde, nitric oxide), reproductive hormones (testosterone, follicle-stimulating hormone, luteinizing hormone), testicular function markers (alkaline phosphatase, acid phosphatase, glycogen, protein), testicular cortisol and cholesterol, histological structure, body and testis weights, and sperm parameters (concentration, total count, motility profile) were assessed.

**Results:**

Heat exposure significantly reduced antioxidant defenses (superoxide dismutase, catalase, reduced glutathione), increased malondialdehyde and nitric oxide levels, and disrupted reproductive hormones (decreased testosterone with increased follicle-stimulating and luteinizing hormones). Heat stress also decreased alkaline and acid phosphatase activities, glycogen, and protein levels, increased testicular cortisol and cholesterol, induced histological damage, and reduced body and testis weights. Sperm quality was impaired, with lower sperm concentration, total count, and fast motility, and higher slow and non-motile sperm fractions. Vitamin C co-treatment partially attenuated these effects, improving catalase activity, *Nrf-2* levels, glycogen, testosterone, alkaline and acid phosphatase, and lessened lipid peroxidation.

**Conclusion:**

Repeated heat exposure induced oxidative stress, hormonal imbalance, impaired sperm quality, and structural testicular injury in Wistar rats. Vitamin C provided partial protection against heat-induced testicular dysfunction, supporting its potential role as an adjunct antioxidant strategy; further studies should evaluate additional antioxidants and include gene expression analyses to clarify mechanisms and fertility outcomes.

## INTRODUCTION

Infertility affects millions of people of reproductive age worldwide. Estimates suggest that between 48 million couples and 186 million individuals live with infertility globally. Infertility in males could result from lifestyle and environmental factors, one of such factors is heat stress. Climate change is a progressive phenomenon that threatens sea level elevations, crop failure and famine, global rainfall patterns, changes to plant and animal populations, and serious health effects (Khan *et al.,* 2020). Climate change has demonstrable effects on health and standard bodily processes since weather and human health are intimately linked. Every year, many people lose their lives because of extremes in temperature. In addition to “excessive aggressiveness,” fatigue, and the inability to focus, heat may also lead to heat exhaustion, heat stroke, heat stress, and even death if it persists for a significant duration of time (Shahat *et al*., 2020). Heat stress leads to an increase in scrotal temperature by impairing the scrotum’s capacity to control its temperature. According to some reports, heat stress has an impact on epididymal sperm, sperm count, sperm motility, and overall sperm quality. Additionally, the spermatogenic process and the generation of male sex hormones are claimed to be affected. Due to its effects on male reproductive endocrinology and other contributing variables, it is one of the main causes of male infertility (Shahat *et al*., 2020; Thanh *et al*., 2020). The testis is the male sex organ responsible for the synthesis of testosterone and spermatocytes through the physiological action of various cells such as Leydig cells, germ cells, peritubular cells and Sertoli cells. The testis is an immune-privileged organ; therefore, several pathological conditions can induce oxidative stress and inflammation, two major causes of male infertility. Testosterone, the main androgen produced by the testes, is synthesized in Leydig cells, primarily under the influence of luteinizing hormone (LH). LH is produced by the anterior pituitary, which, along with follicle-stimulating hormone (FSH), constitutes the hypothalamic-pituitary-testes axis. The hypothalamus releases gonadotropin releasing hormone (GnRH), which stimulates the anterior pituitary to secrete LH and FSH. LH then acts on Leydig cells to promote testosterone synthesis (Nassar & Leslie, 2018; Tiwana & Leslie, 2022).

Due to rising global temperatures, heat Stress is becoming more common and severe (Huang *et al*., 2023; Shahat *et al*., 2020). Exposure to heat stress triggers an overproduction of reactive oxygen species (ROS) and free radicals, leading to oxidative stress, a condition marked by an imbalance between oxidants and antioxidants. This imbalance affects critical cellular components, such as nucleic acids and proteins. The antioxidant defense system, which includes enzymes like superoxide dismutase (SOD), catalase (CAT), and non-enzymatic antioxidants like vitamins E and C, along with the regulatory protein Nrf2, helps counteract ROS. High temperatures can reduce antioxidant enzyme production and lower levels of reproductive hormones, linking oxidative stress to male infertility (Ajeigbe *et al*., 2022; Ngoula *et al*., 2020; Xiong *et al*., 2020; Kumar *et al*., 2016). According to reports, higher temperatures in the environments have an impact on male reproduction. Most elements of mammalian reproductive function are very susceptible to heat stress. Among these are errors in spermatogenesis. These harmful consequences are brought on by either the hyperthermia brought on by heat stress or the physiological changes the heat-stressed animal makes to control body temperature. ROS production is boosted, which has several consequences on gametes and the developing embryo when the temperature is high (Huang *et al*., 2023; Ngoula *et al.,* 2020). The testes are fixed in a scrotum outside the body cavity in most animals, causing the intratesticular temperature to be relatively lower than the body’s core temperature. It has also been hypothesized that the scrotum evolved because of the need for low temperatures, either for spermatogenesis, sperm preservation, or to reduce gamete DNA mutations (Fan *et al*., 2017; McManus *et al*., 2020; Ngoula *et al.,* 2020). A lower temperature in the scrotum than in the body is required for an efficient spermatogenic process. An abnormally high scrotal temperature is one factor in male infertility; as the temperature rises, the quality of the sperm steadily degrades (Durairajanayagam *et al*., 2015; Gao *et al*., 2022). Prior research has shown that heat stress lowers sperm motility and density. In a study done on bulls, heat stress induces dysplasia and lowers sperm quality and libido (Durairajanayagam *et al*., 2015; McManus *et al*., 2020, Thanh *et al.,* 2020). Scrotal heat stress (SHS) triggers several processes in the testes, including the heat shock response, the oxidative stress response, DNA repair, cell cycle checkpoints, apoptosis, and cell death. Scrotal heat stress interrupts spermatogenesis in male mice. Heat stress also affects spermatogonia germ cells, which leads to a decline or disappearance of them in the seminiferous tubules (SHS) may lead to histopathological cellular structural disruptions and an elevated apoptotic rate. Elevated testicular temperatures have been linked to issues with spermatogenesis and steroidogenesis, which may lead to issues with fertility such as germ cell death. Testes have a variety of mechanisms that are triggered upon exposure to SHS, including heat shock response, DNA repair, oxidative stress response, apoptosis, and cell death (Lin *et al*., 2015; Thanh *et al.,* 2020; Zhang *et al*., 2015).

Antioxidants are substances that prevent or slow the oxidation of other molecules, reducing oxidative stress, DNA mutations, and cellular damage. They neutralize reactive oxygen species (ROS) and free radicals, which can cause harm to cells. Vitamin C (ascorbic acid), a water-soluble antioxidant, plays a vital role in protecting cells under heat stress by neutralizing free radicals and preventing oxidation of cell membranes (Santos-Sánchez *et al*., 2019; Adwas *et al*., 2019; Attia *et al*., 2017). This research, therefore, seeks to investigate the effect of heat stress on rats’ testicular function and the possible ameliorative potential of ascorbic acid.

## MATERIALS AND METHODS

### Chemical and reagents

All chemicals and reagents were of analytical grade.

### Experimental animals

All animal procedures in this study were carried out in compliance with the institution’s principles and guidelines, as stated in the guidelines for the use and care of laboratory animals by the National Institute of Health (NIH). Ethical approval was received from Landmark University’s ethical committee. Twenty-four (24) healthy male Wistar rats of *Rattus norvegicus* strain with an average weight of 140g were used. The rats were given 7 days for acclimatization with free access to food and water. They were housed in clean, decontaminated wooden boxes with softwood beddings. The cages had a source of heat which were two (2) 100 watts bulbs fixed on opposite sides inside the cages.

### Experimental design

The rats were distributed randomly into four (4) groups of six (6) rats each. The treatments were administered as follows: Group 1 (control) no exposure to heat or fed ascorbic acid; Group 2 was exposed to heat (40^o^C for 4 hours); Group 3 was exposed to heat (40^o^C for 4 hours every day and fed 50 mg/kg body weight of vitamin C); and Group 4 was fed 50 mg/kg body weight of ascorbic acid. The rats were exposed to heat and administered ascorbic acid for 28 days. Anesthetization was induced with mild diethyl ether; the rats were sacrificed, and testis were harvested into plain sample bottles. The isolated testicular tissues were homogenized with sucrose solution and centrifuged for 10 min at 4^o^C at 5,000 rpm in a refrigerated centrifuge, and the resulting supernatants were collected in fresh sample bottles and stored in a freezer for assays.

### Redox parameters

A series of biochemical assays on rat testis were performed. The antioxidant enzymes analyzed include superoxide dismutase (SOD) activity using the protocol described by Misra & Fridovich (1972) and the activity of catalase (CAT) was determined following the protocol by Beers & Sizer (1952). The determination of reduced glutathione was by the principle stated by Jollow *et al*. (1974). Lipid peroxidation level was carried out using the protocol by Yeo *et al*. (1994). Total protein was determined using the biuret method following the protocol by Gornall *et al*. (1949). Nitric Oxide (NO) was determined following the protocol of Adeyemi *et al*. (2018). Alkaline phosphatase activity was determined following the protocol described by Chapin *et al*. (1987). Acid phosphatase was assessed by the procedure described by Oshiegbu (2022). Testicular glycogen was determined using the procedure of Babaei *et al*. (2021). Testicular cholesterol was determined using the procedure of Rotenberg & Christensen (1976).

### Assessment of the expression levels of nuclear erythrode factor 2, related factor (Nrf-2), hormones, and sperm parameters

A series of hormonal assays were performed. The following hormones, serum testosterone, serum follicle-stimulating hormone (FSH), and serum luteinizing hormone (LH) were assessed using the commercial ELISA kits (Elabscience, Houston, Texas, USA). In addition, the *Nrf-2* expression level was determined using a commercial ELISA kit. All the ELISA kits use the sandwich ELISA principle. Using the optical microscope with a 400x magnification, Sperm motility was assessed, and sperm abnormalities were measured with sperm smears on clean slides. Following the protocol described by Babaei *et al*. (2021), semen analysis was assessed. Testicular cortisol was assayed using the cortisol kit from Calbiotech Inc.

### Histopathology

After being preserved in Bouin’s solution, the fixated rat testis was subjected to standard techniques for histological investigation. The tissues were cleaned in xylene and dehydrated in ethanol before being preserved in paraffin wax. Hematoxylin and eosin (H & E x10) were used to stain the preserved tissue before the slices were cut into sections using a microtome to a thickness of 4-5 m. Unaware of the therapies, a pathologist examined the tissue under a light microscope (Olympus CH; Olympus, Tokyo, Japan).

### Statistical analysis

The experimental data were analyzed using one-way ANOVA (GraphPad Software Inc., San Diego, CA, USA) (SEM). Data were expressed as the mean of six replicates ± standard error of mean except otherwise stated The Tukey’s multiple comparison tests were performed. Mean values were deemed significant at a significance value of 0.05.

## RESULTS

### Effects of heat exposure and vitamin C on redox parameters

**Figure 1:**
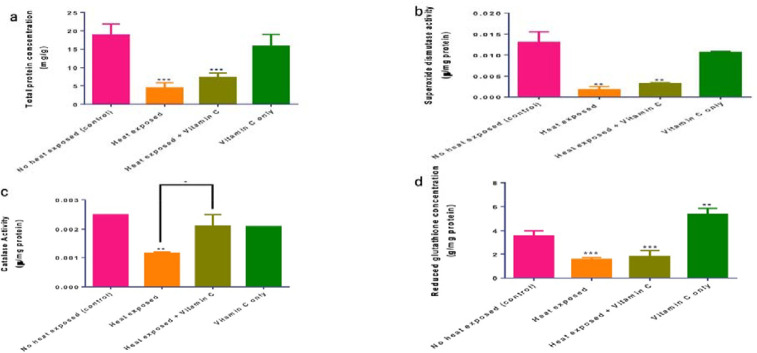
Effect of heat stress and vitamin C on rat testicular. testicular protein (a), superoxide dismutase (b), catalase (c) and reduced glutathione (d). Values are presented as mean±SD, n=6.

The effect of heat and vitamin C on redox parameters is presented in Figure 1. When compared to the control, heat exposed group and heat-exposed + vitamin C group had significantly decreased testicular protein concentration, SOD activity and testicular GSH concentration (Fig. 1a, 1b and 1d). The heat-exposed group had significantly decreased testicular CAT activity compared to the control, there was a significant increase in the CAT activity of the heat-exposed + vitamin C group when compared to the heat-exposed group (Fig. 1c).

**Figure 2:**
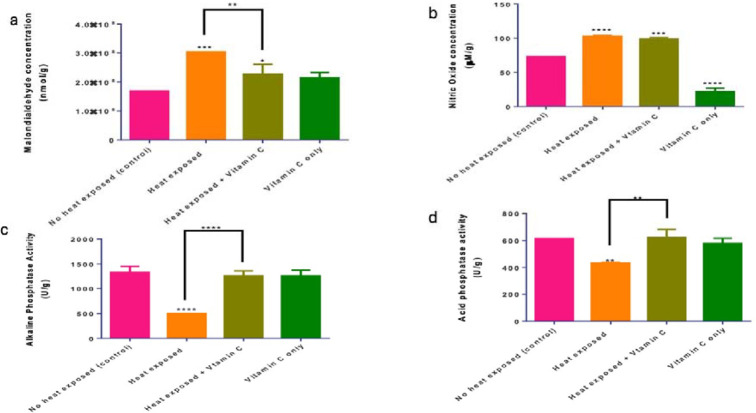
Effect of heat stress and vitamin C on rat testicular. Malondialdehyde (a), nitric oxide (b), alkaline phosphatase (c) and acid phosphatase(d). Values are presented as mean±SD, n=6. Data with an asterisk (s) are significantly different compared to the control at *p*≤0.05.

When compared to the control group, heat-exposed group and heat-exposed + vitamin C group had significantly increased testicular MDA concentration, significantly higher testicular NO concentrations and a significant decrease in the NO concentration of the vitamin C-only group when compared to the control. A significant decrease in the MDA concentration of the heat-exposed + vitamin C group when compared to the heat-exposed group (Fig. 2a and 2b). When compared to the control, heat-exposed group had significantly decreased testicular ALP activity and testicular ACP activity and increase in the ACP and ALP activity of the heat-exposed + vitamin C group when compared to the heat-exposed group (Fig. 2c and 2d).

**Figure 3:**
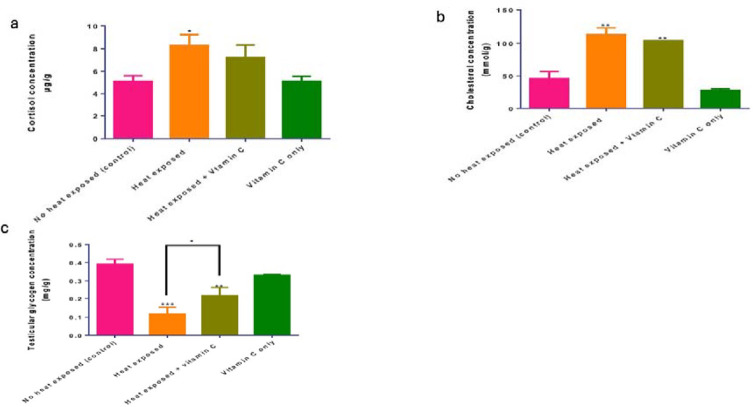
Effect of heat stress and vitamin C on rat testicular. Cortisol (a), cholesterol (b), and glycogen (c). Values are presented as mean±SD, n=6. Data with an asterisk (s) are significantly different compared to the control at *p*≤0.05.

When compared to control, the heat-exposed had significantly higher testicular cortisol concentration and no significant change in the cortisol concentration of the heat-exposed + vitamin C group when compared to the heat-exposed group (Fig. 3a). The heat-exposed and the heatexposed + vitamin C group had significantly high testicular cholesterol concentrations compared to the control and no significant change in the cholesterol concentration of the heat-exposed + vitamin C group when compared to the heat-exposed group (Fig. 3b). The heat-exposed and heatexposed + vitamin C groups had significantly lower testicular glycogen concentrations compared to the control and a significant increase in the glycogen concentration of the heat-exposed + vitamin C group when compared to the heat-exposed group (Fig. 3c).

**Figure 4:**
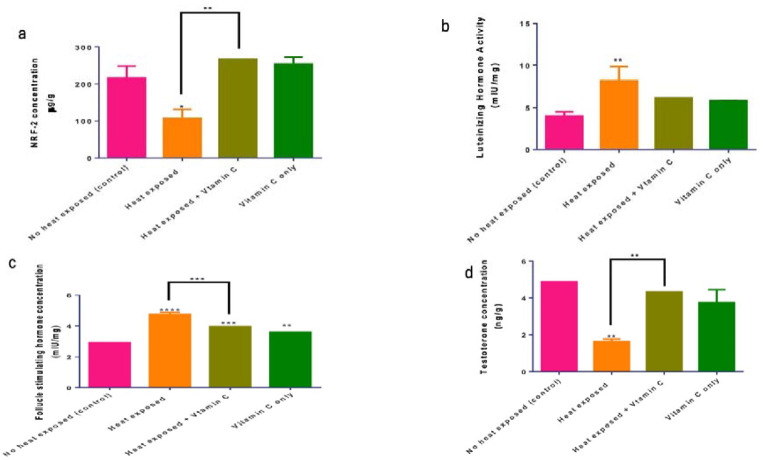
Effect of heat stress and vitamin C on rat serum. Nuclear factor erythroid 2-related factor 2 (Nrf-2) (a), luteinizing hormone (b), follicle stimulating hormone (c) and testosterone (d). Values are presented as mean±SD, n=6. Data with an asterisk (s) are significantly different compared to the control at *p*≤0.05.

**Effects of heat exposure and vitamin C on nuclear erythrode factor 2, related factor 2 (Nrf-2), hormones, and sperm parameters**When compared to the control, the heat-exposed group had significantly decreased testicular Nrf-2 concentration and a significant increase in the *Nrf-2* concentration of the heat-exposed + vitamin C group when compared to the heat-exposed group (Fig. 4a). The heat-exposed group had significantly increased serum LH concentration compared to the control and no significant change in the serum LH concentration of the heat-exposed + vitamin C group when compared to the heat-exposed group (Fig. 4b). The heat-exposed, heat-exposed + vitamin C and the vitamin C-only group had significantly higher serum FSH concentrations compared to the control and a significant decrease in the FSH concentration of the heat-exposed + vitamin C group when compared to the heat-exposed group (Fig. 4c). The heat-exposed group had significantly decreased serum testosterone concentration compared to the control. The results also show that there was a significant increase in the serum testosterone concentration of the heat-exposed + vitamin C group when compared to the heat-exposed group (Fig. 4d).

### Histopathology

**Figure 5:**
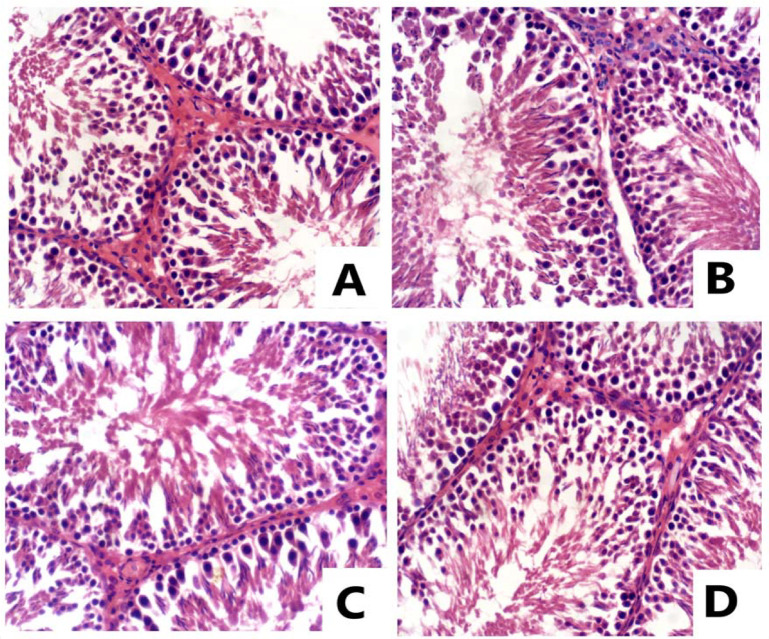
Effect of heat and vitamin C on testicular tissue histopathological structure. Where: **Group A=**No heat exposed (control) group; **Group B=**Heat exposed group; **Group C=**Heat exposed + vitamin C group; and **Group D=**Vitamin C only.

When compared to the control group, heat-exposed group shows a disruption in the normal physiology of testicular tissues (Figure 5).

### Semen analysis

Quantitative analysis of sperm parameters (Table 1) revealed that heat exposure significantly reduced semen concentration, total sperm count, and percentage of fast motile sperm compared with the control group (*p*<0.05). Heat exposure also significantly increased the percentages of slow and non-motile sperm cells, as well as sperm morphological abnormalities. Administration of vitamin C to heat-exposed rats resulted in modest improvements in selected sperm parameters compared with the heat-only group, although values did not fully return to control levels. Rats treated with vitamin C alone showed sperm parameters comparable to control (Table 1).

**Table 1:** Effects of heat and vitamin C on rat sperm parameters. (each value is represented as a mean of six replicates).

Semen	GROUP			
	No heat exposed (control)	Heat exposed	Heat exposed + vitamin C	Vitamin C only
Concentration (×10^6^/mL)	91.00±11.50	61.00±5.50 ^***^	61.00±0.50^***^	90.50±5.50
Total sperm Count (×10^6^/total volume)	136.92±15.20	64.70±22.30^***^	74.16±10.37 ^***^	109.17±21.59
% Fast Sperm	47.50±4.00	30.00±0.00^*^	30.00±0.00^*^	32.00±0.00^*^
% Slow Sperm	17.50±0.30	40.00±0.50^**^	30.00±0.10	27.50±0.40
% Non-Motile Sperm	27.50±0.10	52.50±0.21^*^	40.00±0.00	25.00±0.10
Normal	65.00±5.2	57.50±0.0	60.00±2.4	67.5±0.23
Head Distorted	7.50±0.0	10.00±2.1	10.00±3.1	10.00±0.9
Neck Distorted	5.00±0.1	12.50±0.8	12.50±1.1	10.00±0.1
Tail Distorted	10.50±1.2	22.50±4.23^**^	20.00±2.1^**^	10.00±0.9

Values are expressed as mean±SEM (n=6). One-way ANOVA followed by Tukey’s post hoc test was used for statistical comparisons.

* *p*<0.05 *vs*. control,

** *p*<0.01 *vs*. control,

*** *p*<0.001 *vs*. control.

### Body weight index

As shown in Table 2, heat exposure resulted in a significant reduction in final body weight and testicular weight compared with the control group (*p*<0.05). Although vitamin C administration slightly improved final body weight in heat-exposed rats, values remained lower than control. The organ-to-body weight ratio showed no marked restoration with vitamin C treatment.

**Table 2:** Effects of heat and vitamin C on rat body and organ weight index. (each value is represented as the mean of three replicates).

Weight	GROUP			
	No heat exposed (control)	Heat exposed	Heat exposed + vitamin C	Vitamin C only
Initial Body Weight	178.67±12.4	167.50±22.1	168.00±19.67	176.17±12.30
Final Body Weight (After Heat Exposure)	245.67±3.0	193.00±12.8	206.5±20.3	226.33±13.12
Testis Weight	1.35±0.45	1.19±0.12	1.22±0.23	1.25±0.71
Organ-Body Weight Ratio	5.50×10^-3^	6.17×10^-3^	5.91×10^-3^	5.52×10^-3^

Values are expressed as mean ± SEM (n=6). One-way ANOVA followed by Tukey’s post hoc test was used for statistical comparisons.

## DISCUSSION

Exposure to heat stress triggers an overproduction of reactive oxygen species (ROS) and free radicals, leading to oxidative stress, a condition marked by an imbalance between oxidants and antioxidants. Body weight index has been established as a measure of health. Significant loss of weight has been shown to indicate deterioration in the general health of an organism (Qu *et al*., 2021). This study showed that rats had a significant decrease in body weight after exposure to heat, which suggests that heat deteriorated the general health of the rats (Attia *et al*., 2017). This result is synonymous with the result observed by Attia *et al*. (2017) where exposure to heat caused a reduction in the body weight of rats exposed to heat. Protein functions as a structural part and a fundamental building block of cells, tissues, and enzymes, it also serves as a source of energy (Laddomada *et al*., 2016). The significant decrease in the protein concentration in the heat-exposed group and heat-exposed + vitamin C group compared to the control group could have occurred because heat exposure denatures protein and interferes with the synthesis of protein and various enzymes (Hosseindoust *et al*., 2020) and this is in correlation with the results of the study done by Setchell (2018) where heat decreased secretion of androgen binding protein of Sertoli cells in the testis.

Superoxide dismutase (SOD) an antioxidant enzyme that scavenge superoxide anion (Ali *et al*., 2020). showed decrease activity in both the heat-exposed and heat-exposed + vitamin C group when compared to control. Previous studies have shown heat induced enzyme denaturation (Firmansyah & Argosubekti, 2020). Vitamin C could not mitigate this effect, which is consistent with the work of Kumar et al. (2016) where heat exposure decreased the SOD concentration in male Wister rats. Catalase (CAT), an antioxidant enzyme that neutralizes hydrogen peroxide to water and oxygen (Nandi *et al.,* 2019), showed decreased activity in the heat-exposed group when compared to the control group, suggesting accumulation of hydrogen peroxide which could be an indication of lipid peroxidation and oxidative stress (Alhaithloul, 2019). Vitamin C effectively mitigated this reduction which is consistent with the work of Kumar *et al*. (2016) where heat exposure decreased the CAT levels in male Wistar rats. Glutathione (GSH), a key ROS scavenger (Gaucher *et al.,* 2018), showed significantly decreased concentrations in both the heat-exposed and heat-exposed + vitamin C groups compared to the control. this could predispose the rats to oxidative stress because of the unavailability of GSH which could increase ROS (Kerchev & Van Breusegem, 2022). Vitamin C was not efficient in restoring GSH concentration in heat-exposed rats. This is in correlation with the work of Xiong *et al*. (2020). Malondialdehyde (MDA) a key marker of lipid peroxidation (Morales & Munné-Bosch, 2019), showed significant increase in both heat-exposed and heat-exposed + vitamin C group compared to the control suggesting lipid peroxidation (Morrell, 2020). Vitamin C was effective in reducing lipid peroxidation levels which is in correlation with the work of Xiong *et al*. (2020) where exposure to heat caused an increase in lipid peroxidation and alpha lipoic acid was able to reduce lipid peroxidation and MDA concentration. Nitric oxide radical (NO•) though physiologically significant, contributes to oxidative stress when combined with other ROS (Pizzino *et al.,* 2017). Heat-exposed group significantly increased NO concentration when compared to the control, indicating ROS production (Rahman & Rahman, 2021). Also, vitamin C-only group had significantly lower NO concentration when compared to the control which could be an indication of the antioxidant abilities of vitamin C. The results of this study are similar to the study of Gharibi *et al*. (2020) on industry workers where exposure to heat caused an increase in ROS production. Nuclear factor erythroid 2-related factor 2 (*Nrf-2*), a key regulator of cellular defense against oxidative stress, showed significantly lower levels in heat-exposed group compared to control, indicating impaired *Nrf-2* function due to heat exposure (Chambel *et al*., 2015). Vitamin C effectively restored *Nrf-2* levels, consistent with the study of Malyar *et al*. (2021) where the heat-stressed group experienced a decrease in *Nrf-2* concentration. Alkaline phosphatase (ALP) and acid phosphatase (ACP), markers of testicular function, showed significantly decreased activities in the heat-exposed group compared to controls, indicating impaired testicular function due to heat exposure (Owumi *et al*., 2020; Firmansyah & Argosubekti, 2020). Vitamin C mitigated these effects, restoring ALP and ACP activity. These findings align with Saleh *et al*. (2022), who observed reduced ALP levels in heat-exposed industry workers.

Cortisol is the major glucocorticoid in humans, it functions primarily to stimulate gluconeogenesis and activate anti-stress and anti-inflammatory pathways, it is also known as the “stress hormone” (Cole et al., 2019). heat-exposed group compared to the control. This could be an indication that heat exposure predisposed the rats of this group to stress which led to the release of this stress hormone (Bagath *et al*., 2019). Vitamin C was not effective in reducing stress levels which is consistent with studies by Oluwagbenga *et al*. (2022). Glycogen, an energy reserve in testicular cells, showed significant decrease in both the heat-exposed and heat + vitamin C group when compared to the control, suggesting that heat exposure depleted the energy reserves of this group of rats (Gonzalez-Rivas *et al*., 2020). Vitamin C effectively restored glycogen level of rats exposed to heat, consistent with Attia *et al*. (2017) who observed that vitamin C and E mitigated heat-induced glycogen depletion in broilers. Cholesterol is the primary precursor of steroid hormones, primarily testosterone. increase in cholesterol levels in the heat-exposed and the heat-exposed + vitamin C group, suggesting steroidogenesis might have been modulated and therefore causing an accumulation of the precursor (Alani *et al*., 2021). Vitamin C was not effective in mitigating the effects of heat exposure on steroidogenesis. This is in correlation with the study done by Attia *et al*. (2017) where heat stress caused an increase in the cholesterol levels of broilers. Testosterone, the primary sex hormone, significantly decreased in the heat-exposed group when compared with the control group. Previous studies have shown that low testosterone could lead to male hypogonadism, it could also affect male reproductive functions such as sperm production and sex drive (Corona *et al.,* 2022). This suggests that exposure to heat impeded the production of testosterone (via steroidogenesis) and vitamin C was able to mitigate this effect (Alani *et al*., 2021). This is in correlation with the work of Xiong *et al*. (2020) where exposure to heat caused a decrease in serum testosterone concentration and alpha lipoic acid was able to raise testosterone concentration. Luteinizing hormone (LH), which controls steroidogenesis in the male reproductive system (Santi *et al.,* 2020), showed a significant increase in the heat-exposed group when compared with the control. Vitamin C was unable to ameliorate this effect. Since the secretion of luteinizing hormone is tightly controlled by the hypothalamic-pituitary-gonadal axis, high levels of luteinizing hormone in the bloodstream may be a result of the decreased sex steroid production from the testis as a result of the negative feedback effect of testosterone (Nedresky & Singh, 2019). This is in correlation with the study of An *et al*. (2020), where heat stress caused an increase in the LH of rats. Follicle-stimulating hormone (FSH), which stimulates spermatogenesis, significantly increased in the heat-exposed group when compared to the control. This may cause decreased testicular activity resulting in an alteration of the normal feedback mechanism between the testes and the hypothalamic-pituitary axis, through impairment of Sertoli cells, and decreased inhibin secretion (Airaodion *et al.,* 2019). This effect may be a result of the negative feedback effect from the low levels of testosterone (Nedresky & Singh, 2019). However, vitamin C was effective in ameliorating this effect of heat. The results of An *et al*. (2020) corroborate these results.

The observed reduction in sperm concentration and motility may be attributed to oxidative damage to germ cells and impaired steroidogenesis, as evidenced by reduced *Nrf-2* level, decreased antioxidant enzyme activities, and lowered testosterone concentration. Since spermatogenesis is highly dependent on both redox balance and androgen support, disruption of these pathways likely contributed to the observed sperm abnormalities..”

Histopathological examination revealed disrupted testicular tissue physiology in the heat-exposed group, indicating impaired spermatogenesis, consistent with findings by Thanh *et al*. (2020) in heat-stressed mice. Semen analysis showed reduced semen volume, total sperm count, and fast sperm percentage, alongside increased slow and non-motile sperm, suggesting heat-induced spermatogenesis disruption. These results align with Huang *et al*. (2023), who reported decreased sperm motility, density, and volume, as well as increased sperm malformation under heat stress.

## CONCLUSION

The findings indicate that exposure to heat significantly decreased the activities of antioxidant enzymes namely catalase, superoxide dismutase and increased the nitric oxide concentration but reduced the nuclear factor *Nrf-2*. It also significantly increased lipid peroxidation, but reduced the testicular function indices like alkaline phosphatase, acid phosphatase, glycogen and protein, while increasing cholesterol concentration. It affected the reproductive hormone concentration, increasing follicle-stimulating hormone and luteinizing hormone but reducing testosterone. Exposure to heat also affected the semen function parameters and disrupted the histopathology structure of the testicular tissues. Vitamin C was partially effective in ameliorating these effects, ameliorating the effect on catalase activity, *Nrf-2*, glycogen testosterone, alkaline phosphatase, acid phosphatase concentrations and decreasing lipid peroxidation levels. Future research should explore other antioxidants and possible phytochemicals with antioxidant potentials and carry out gene expression analysis to determine fertility level.

## Abbreviations:

CAT: catalase; GSH: reduced glutathione; FSH: follicle-stimulating hormone; MDA: malondialdehyde; NO: Nitric oxide; SOD: superoxide dismutase; LH: luteinizing hormone; GnRH: gonadotropin-releasing hormone; ROS: reactive oxygen species; SHS: Scrotal heat stress.
